# Robust LSPR Sensing Using Thermally Embedded Au Nanoparticles in Glass Substrates

**DOI:** 10.3390/nano11061592

**Published:** 2021-06-17

**Authors:** Nuno M. Figueiredo, Ricardo Serra, Albano Cavaleiro

**Affiliations:** 1 CEMMPRE—Department of Mechanical Engineering, University of Coimbra, Rua Luís Reis Santos, 3030-788 Coimbra, Portugal; ricardo.serra@dem.uc.pt (R.S.); albano.cavaleiro@dem.uc.pt (A.C.); 2 LED&MAT—Instituto Pedro Nunes, Rua Pedro Nunes, 3030-199 Coimbra, Portugal

**Keywords:** localized surface plasmon resonance (LSPR) sensing, Au nanoparticles (NPs), thermal embedding, thermal dewetting

## Abstract

The poor adhesion and chemical and thermal stability of plasmonic nanostructures deposited on solid surfaces are a hindrance to the longevity and long-term development of robust localized surface plasmon resonance (LSPR)-based systems. In this paper, we have deposited gold (Au) nanolayers with thicknesses above the percolation limit over glass substrates and have used a thermal annealing treatment at a temperature above the substrate’s glass transition temperature to promote the dewetting, recrystallization, and thermal embedding of Au nanoparticles (NPs). Due to the partial embedding in glass, the NPs were strongly adherent to the surface of the substrate and were able to resist to the commonly used cleaning procedures and mechanical adhesion tests alike. The reflectivity of the embedded nanostructures was studied and shown to be strongly dependent on the NP size/shape distributions and on the degree of NP embedding. Strong optical scattering bands with increasing width and redshifted LSPR peak position were observed with the Au content. Refractive index sensitivity (RIS) values between 150 and 360 nm/RIU (concerning LSPR band edge shift) or between 32 and 72 nm/RIU (concerning LSPR peak position shift) were obtained for the samples having narrower LSPR extinction bands. These robust LSPR sensors can be used following a simple excitation/detection scheme consisting of a reflectance measurement at a fixed angle and wavelength.

## 1. Introduction

Conductive materials in the form of nanoparticles (NPs) are shown to exhibit size-, shape-, and material- dependent properties that are not present in bulk, such as increased chemical reactivity and selectivity [1] and unusual optical [2,3,4], thermal [5,6], mechanical [7], and magnetic [8] properties. Gold (Au) NPs, in particular, have attracted increasing attention over the past decades due to their unique properties that make them attractive for use in several optical, electronic, decorative, catalytic, biological, and medical applications [4,9]. One of the most interesting properties of Au NPs is their ability to support surface plasmons (SPs), collective oscillations of free electrons after excitation by external incident light. Since these plasmons oscillate locally around the NPs, they are called localized surface plasmon resonances (LSPRs). At the resonance frequency, the electric field near the NPs’ surface is greatly enhanced and the optical extinction (absorption + scattering) is considerably increased. The shape and positioning of the LSPR extinction bands is highly dependent upon the NPs’ size, shape, interparticle distance, and local refractive index [2,3,4,9]. The latter dependency enables the use of Au NP systems as optical transducers for detecting and quantifying chemical or biological analytes in the surrounding environment of the NPs, by means of monitoring changes in the SP extinction bands.

NPs can be produced by chemical or physical means [4]. Sputtering-based techniques are extremely attractive and are commonly used in industrial and research environments due to their inherent advantages such as: good control over the NP properties, non-use of solvents or harmful stabilizers, low cost, large area deposition capabilities, simple and fast deposition processes, consistent quality and reproducibility [4,10]. The main nanocomposite configurations being used nowadays for most applications consist in forming NPs of specific size-, shape-, and lattice- distributions either at the surface or embedded within a host matrix, or a combination of both [4]. Challenges arise in the case of NPs supported on a surface due to several factors: poor adhesion is often expected and aging takes place, even at room temperature, due to limited chemical [11,12] and thermal [13,14] stability of the NPs, progressively degrading the optical properties necessary for guarantying the longevity and long-term development of the LSPR nanostructures.

Three main strategies were suggested for stabilizing plasmonic NPs over surfaces: (i) application of ultrathin inert overlayers encapsulating the NPs [15,16,17]; (ii) utilization of metallic [18,19,20] or organic [19,21,22] coupling underlayers; (iii) thermal embedding of NPs on the surface of amorphous materials [22,23,24,25]. The first two approaches have shown to induce optical and/or chemical interference. The third approach seems to be the most promising and less explored one of the three. Thermodynamically, the immersion of a rigid metal nanoparticle on a soft matrix material can happen when the surface tension of the NP/air interface is greater than the sum of the surface tensions at the matrix/air and NP/matrix interfaces. When considering NP embedding into a solid-state amorphous material, the creation of a void (indentation) is required, i.e., the Gibbs free energy of the system must be changed promoting the work of adhesion. This energy can be supplied by external heating at temperatures near glass transition temperature (Tg) of the amorphous material [26,27]. Very few publications exist on this topic dealing with non-polymeric substrates, mostly being led by Rubinstein’s research group [22,23,24,25]. They were mainly focused on the thermal evaporation of Au thin films on glass substrates, with thicknesses below and above the percolation limit, and their annealing at temperatures close to the substrate’s Tg. So far, studies on sputtered Au thin films and their subsequent thermal embedding on glass substrates have not been performed, to our best knowledge. Moreover, most studies on this subject are only focused on monitoring the transmittance spectrum of the LSPR bands. The use of reflectance configuration for sensing experiments could be a more interesting approach to use due to its simpler, more portable, and cheaper configuration.

In this study we have sputter deposited Au nanolayers with thicknesses above the percolation limit over glass substrates and have thermally annealed them at a temperature above the substrate’s Tg to promote dewetting, recrystallization and thermal embedding of Au NPs in the glass surface. Highly stable and robust plasmonic nanostructures were obtained in this way, allowing a full-time cyclic operation of the LSPR sensor.

## 2. Materials and Methods

### 2.1. Au NPs Deposition and Thermal Annnealing above Tg

Three Au nanolayers of different nominal thickness (4 nm, 8 nm and 12 nm) were sputter deposited using a pure (99.999%) Au cylindrical target (50.8 mm in diameter by 3 mm in thickness) in a pure Ar (99.999%) atmosphere. The Ar flux in the inlet was set to 30 sccm, corresponding to a constant deposition pressure of 0.3 Pa. The target-to-substrate distance was maintained at 12 cm and the rotation speed of the substrate holder was set to 30 rpm. The glass substrates (microscope glass slides from Histo Star, Tg = 557 °C) were kept at floating potential. A d.c. power supply (Advanced Energy Pinnacle Plus) was used with a constant power of 50 W during deposition. The deposition chamber was pumped with a rotary (Pfeiffer Vacuum DUO 20 M, pumping speed 20 m^3^/h) and diffusion (BOC Edwards Diffstak 160/700, pumping speed 760 l/s) pumps to a base pressure below 5 × 10^−4^ Pa.

After the deposition process, all samples were subject to thermal annealing treatments at 630 °C in a horizontal furnace kept at open atmosphere. The heating ramp was set to 33 °C/min. Two different isothermal periods were used for each sample: 1 h 30 min and 10 h 30 min.

### 2.2. Au NPs’ Dissolution

Au NPs were dissolved from the glass substrates by dipping the samples into freshly prepared aqua regia solution (3:1 HCl:HNO_3_) during 3 min, followed by rinsing with deionized water.

### 2.3. Adhesion and Stability Tests

The strength of the Au NPs adhesion to the glass substrates was evaluated qualitatively using the peel tape adhesion test in accordance to ASTM D3359—Standard Test Methods for Measuring Adhesion by Tape Test. Shortly, a piece of clear Scotch tape (3M) was pressed against the samples’ surface and pulled away. In the case of any poorly adhesive nanolayer or NP configuration, detachment is clearly observable with naked eyes.

In order to further test the mechanical robustness of the samples, 1 cm viton balls were adapted into a scratch tester stylus to perform scratch tests using a progressive load setup up to 15 N. The vertical load rate was set to 100 N/min, whereas the horizontal velocity was set to 30 mm/min. All tests were performed on a CSEM Revetest automatic scratch tester. The stability of the optical signal to immersion in liquids was evaluated by comparing reflectance spectrum before and after an ultrasonic cleaning bath in ethanol for 20 min and drying.

### 2.4. Determination of the Refractive Index Sensitivity (RIS)

Test solutions with variable refractive index (RI) in the range 1.333 to 1.473 were prepared by mixing distilled water (*n* = 1.333) and glycerol (*n* = 1.473) at different ratios (1:0, 1:1; 0:1). The LSPR sensors were hold horizontally on a homemade sample holder and, prior to each test, an o-ring was placed above the samples surface to confine the variable refractive index liquids. Placing three drops of solution was enough to promote the total wetting of the surface being analyzed from the top side by reflectivity measurements. Between measurements, the samples were thoroughly cleaned up using distilled water baths until no change in the reflectance spectrum was observed. The optical reflectance of all samples was analyzed on a Gretagmacbeth X-Rite ColorEye XTH spectrophotometer.

### 2.5. Structure and Surface Morphology

The structure of the Au NPs/nanolayers was analyzed by X-ray diffraction (XRD) on a high-resolution Philips PANalytical diffractometer with Cu Kα_1_ radiation in grazing incidence configuration, with an incidence angle of 2°. The XRD peak parameters, such as peak position, peak intensity, and full width at half maximum (FWHM) were evaluated after peak fitting assuming a pseudo-Voigt function. The FWHM of the peaks was corrected by deconvoluting the instrumental line broadening [28]. The crystallite size was estimated from the (111) peak position and FWHM using the Scherrer equation [29]. The surface morphology of samples was analyzed by atomic force microscopy (AFM) on a Bruker diInnova using a silicon tip with 6 nm of tip radius. Grain size, shape, and height distributions were obtained for each sample using the open source software Gwyddion [30].

## 3. Results and Discussion

Nanometric thin Au layers were deposited over glass slides, using different nominal thicknesses of 4 nm, 8 nm and 12 nm, originating continuous Au nanolayers with varying thicknesses (see Supplementary Figure S1). The samples were thermally annealed at 630 °C—a temperature above the glass transition temperature of the substrates (Tg = 557 °C)—for 1 h 30 min and for 10 h 30 min in order to promote dewetting, recrystallization and thermal embedding of Au NPs in the glass surface. A study of their main properties was performed, including on their structure, Au NP size, shape and surface distribution, and percentage of thermal embedding as a function of Au content and annealing time. Their functional properties, namely the optical reflectivity and the refractive index sensitivity, were also studied in detail.

### 3.1. Structure and Crystallite Size

**Figure 1:**
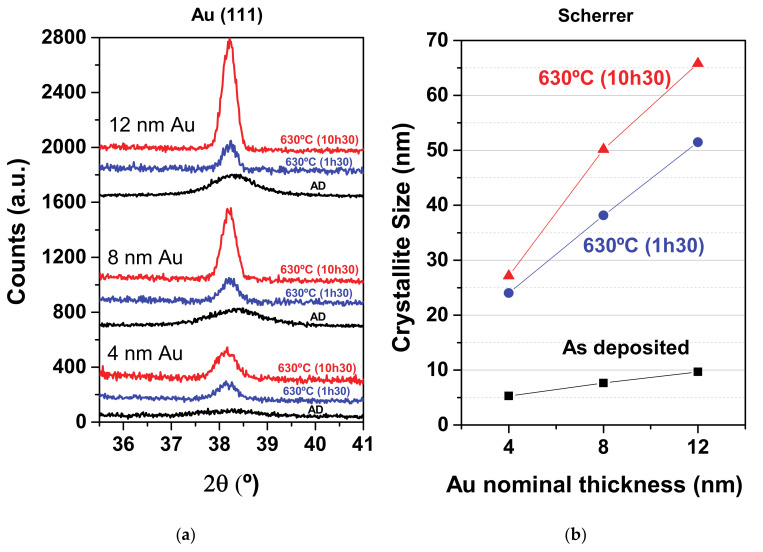
(**a**) XRD patterns of the Au NPs deposited over glass substrates, as a function of the Au nominal thickness and annealing time; (**b**) Au crystallite size estimation using Scherrer equation.

The structure of each sample was studied by XRD, before and after each thermal annealing treatment, as shown in Figure 1a. Broad (111) diffraction peaks of the face centered cubic Au phase were noted in the diffractograms for all as-deposited samples, which slightly increased in crystallinity with the Au content. The crystallinity of the (111) diffraction peaks increased progressively with the annealing time. Therefore, the most crystalline sample was the 12 nm Au nanocomposite after thermal annealing at 630 °C for 10 h 30 min. For each case, the Au crystallite size was estimated using Scherrer equation, as shown in Figure 1b. For the as-deposited samples, the crystallite size correlated well with the nominal thickness of the coatings. Concerning the thermally annealed samples, the crystallite size correlated well with the Au NP total heights observed by AFM (Table 1). NP heights between ~25–70 nm were suggested by this analysis.

### 3.2. Surface Morphology

**Figure 2:**
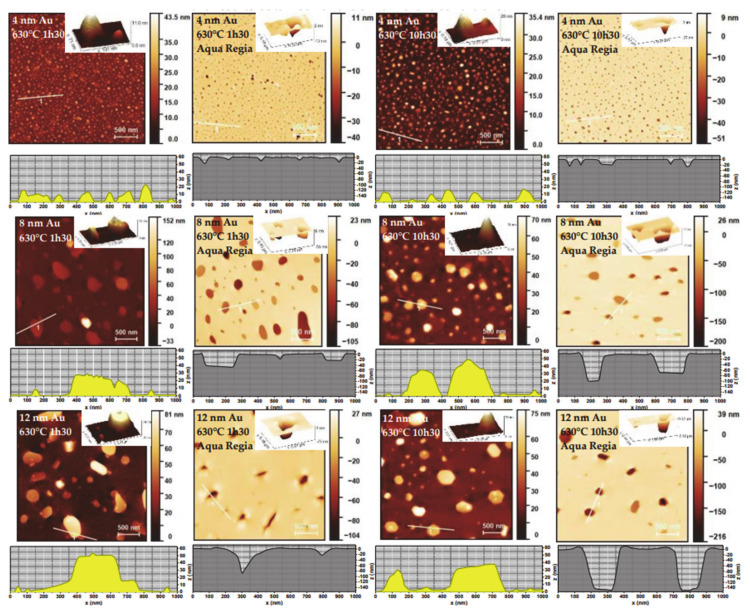
AFM pictures of each thermally annealed sample (at 630°C, for 1 h 30 and 10 h 30), before and after Au dissolution with aqua regia solution. Insets show close-up images in 3D. Selected line profiles are depicted below each AFM image.

The surface morphology of each thermally annealed sample was studied by AFM, before and after Au dissolution in aqua regia solution, in order to infer the size/height of the NPs and their indentation size/depth on the glass surface, respectively. Figure 2 shows the results obtained for the six different conditions.

After carefully analyzing the AFM images, a set of statistical data concerning the average NP diameter and height was obtained using Gwyddion software [30]. For each sample, the average total height of the NPs was calculated by adding the average NP height (obtained before chemical etching) to the average indentation depth (obtained after chemical etching). Distribution curves of the equivalent diameters (diameter of a circle with the same top-view projected area as the NP) can be found in Supplementary Figure S2. Moreover, from the top view images, it was possible to fit ellipses to each NP and study their average aspect ratio AR1. A secondary aspect ratio AR2 was determined relating the minor axis of the fitted ellipses (corresponding to the 1st axis of an ellipsoid) to the total height of the NPs (corresponding to the 3rd axis of an ellipsoidal NP). From these data, the percentage of thermal embedding was calculated. Most data results gathered from statistical analysis are shown in Table 1.

**Figure 3:**
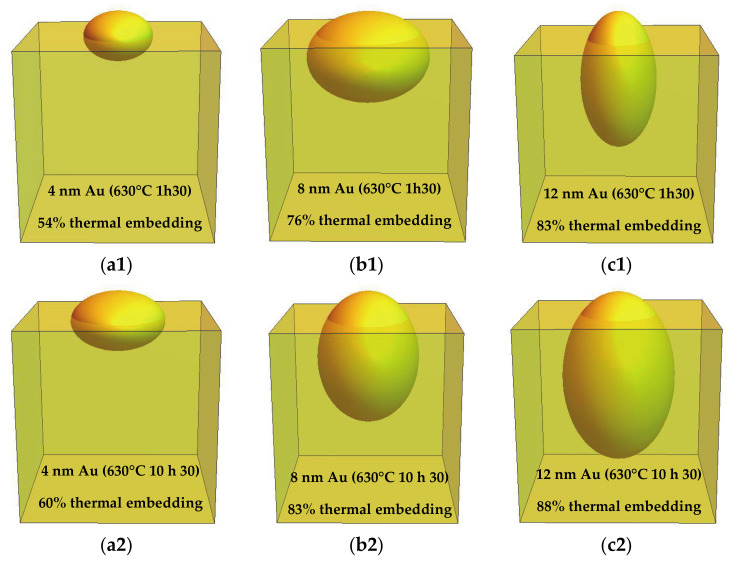
Representative 3D drawing of each sample’s microstructure after thermal annealing treatments at 630 °C: (**a1**) 4 nm Au annealed for 1 h 30; (**b1**) 8 nm Au annealed for 1 h 30; (**c1**) 12 nm Au annealed for 1 h 30; (**a2**) 4 nm Au annealed for 10 h 30; (**b2**) 8 nm Au annealed for 10 h 30; (**c2**) 12 nm Au annealed for 10 h 30. Ellipsoids were used as an approximation for the Au NPs’ shape. The edges of the cubes, representing the glass substrates, measure 100 nm in length. The formation of a glass rim surrounding the NPs is not accounted for in these representations.

The microstructure images shown in Figure 3 can be seen as a general representation of each sample in that they represent the average shape and percentage of thermal embedding based on the overall statistics of the NP populations. However, they should be taken carefully since they don’t have into consideration the volumetric distribution of Au atoms at the glass surface, i.e., larger NPs are statistically in a much lesser number but take the majority of Au atoms in their constitution. Particularly for the 8 and 12 nm Au cases there is a big dispersion of NP sizes due to the presence of a large number of smaller ~40 nm NPs along with a smaller number of very large ~200–500 nm NPs (see Supplementary Figure S2). As a particular example, for the 12 nm Au sample annealed for 10 h 30 min at 630 °C there are 6.3 times more NPs in the 1–100 nm range than there are in the whole 101–500 nm range. However, the total NP volume is 39 times higher in the latter case (larger NP range). This is an important consideration since the optical properties of any plasmonic material scales strongly with the volume of the plasmonic features, namely the light extinction.

The representative 3D microstructure configurations of the NPs over the glass slides were recreated using Wolfram Mathematica software [31] and are shown in Figure 3.

From Figure 3, we can identify two distinct preferential NP embedding orientations: for the 4 nm Au samples and for the 8 nm Au sample annealed for 1 h 30 min the NPs are embedded with their major ellipsoidal axis aligned parallel to the surface of the substrates, whereas for the 8 nm Au sample annealed for 10 h 30 min and for the 12 nm Au samples the NPs are embedded with their major ellipsoidal axis aligned perpendicularly to the surface of the substrates. For both 4 and 12 nm Au cases, both average NP (top-view) size and average NP (cross-sectional-view) total height continue to increase with the annealing time, from 1 h 30 min to 10 h 30 min, whereas in the 8 nm Au case, there is a reduction in the NP (top-view) size and an increase in the (cross-sectional-view) NP total height. The major driving force for solid-state dewetting of thin nanometric layers is to decrease the total energy of all surfaces and interfaces. Parameters such as thin layer thickness, grain size, fraction of open voids at the metal-air interface, deposition technique and alloying all significantly impact on the dewetting process [32,33,34]. In order to minimize the surface area, the NPs’ tendency is to adopt spherical shapes. In this case, an additional thermal embedding mechanism is contributing towards the surface area minimization process. Thus, given enough temperature and/or annealing time, the reshaping of the NPs together with the thermal embedding can take place towards this objective. The transition in NP shape orientation to have the major ellipsoidal axis aligned perpendicularly to the glass surface seems to be the most favorable geometry for protruded NP surface area minimization in the case of NPs of larger volumes.

Just like in previous publications on thermally embedded Au NPs on glass surfaces prepared by evaporation methods [22,23,24,25], we noted the formation of glass rims at the glass/metal interface, as clearly shown in the insets of Figure 2. Most rims were concentric, with variable height along the border of the NP indentations, generally increasing in height with the NP indentations depth. There is still not a clear explanation of the mechanisms of glass rim formation surrounding the metal NPs in high temperature annealing, around the glass Tg, in oxidative atmospheres, e.g., the possibility of thermally induced local chemical interaction between Au and glass remains an open question [25]. However, a simple analogy could be made to the pile-up formed around an indenter in the case of nanoindentation tests [35]. As such, the glass rim formation could be linked to the diffusion of glass atoms from the indentation zones at the moment of accommodating the Au NPs in glass. In the case of nanoindentation the driving force is the mechanical force, whereas in the case of thermal embedding the driving force is the capillary motion (contributing towards the presence of more wavy surfaces without any abrupt discontinuities at the metal/glass interface).

The continued reshaping of Au NPs with annealing time observed in this study contrasts with previous observations of thermally evaporated Au thin films where the NP shape was pretty much unchanging with time after 30 min of thermal annealing at 600–650 °C, while the embedding depth in glass increased [24,25]. Therefore, the solid-state dewetting process of our magnetron-sputtered nanolayers was slower, allowing for a better control over the final microstructure of the plasmonic nanomaterial. This points to a more compact microstructure of our magnetron-sputtered Au nanolayers, having a lower fraction of open voids. In fact, we observed percolation at 4 nm and DC conductivity in all as-deposited samples, whereas in one study for evaporated Au nanolayers the observed threshold for full percolation with DC conductivity was situated at around 10 nm [22]. Thus the initial deposition process and deposition conditions can all impact on the final microstructure of the thermally embedded NPs.

The NP indentation depths increased with both Au content and annealing time. The average NP indentation depth values varied from 14–19 nm, 38–56 nm and 59–81 nm for the 4, 8, and 12 nm Au samples, respectively, with increasing annealing time (corresponding to a ~40% increase from 1 h 30 min to 10 h 30 min), whereas the average NP height values above the glass surface stayed approximately constant at ~12 nm in all samples. These observations, translated into NP thermal embedding percentages, are way larger than the ones observed before in literature for similar Au-glass systems: 54–60%, 76–83% and 83–88% for the 4, 8 and 12 nm Au samples, respectively. Au NP heights of around ~ 20–40 nm and depression depths of ~2.5–7.5 nm were observed by Karakouz et al. [23], resulting in a maximum percentage of thermal embedding of 27%. In another study by the same author [24], Au NP heights of around 26 nm and depression depths of around 27 nm were observed, resulting in 51% of thermal embedding. We could tentatively attribute the larger indentation depths observed in this study to combined differences in the glass chemical composition and in the glass surface roughness promoting a smaller glass surface energy and consequently an increased tendency for NP immersion in order to minimize tension at the interface [27].

Another observation we can make is that although the measured average NP diameter and that of their indentations agrees well in the 4 nm Au case (especially if a deconvolution of tip effects is made following the approach given in [36]), for the 8 and 12 nm Au cases the measured diameter of the Au NP indentations is overall ~50% or ~150% larger than the measured NP diameter, respectively. We can give three main reasons for this discrepancy: (i) due to our AFM tip configuration (tip radius of 6 nm) the smaller-sized NP indentations (<10 nm) were not measured; (ii) the larger NPs were mostly dissolved whereas a fraction of the smaller NPs was not totally dissolved in aqua regia (they appear as small white spots in the AFM images of Figure 2); and (iii) another possibility is that a mild chemical etching of the glass surface could have taken place preferentially at the larger pore openings (i.e., NP indentations). Although we haven’t found any standalone study in literature concerning the chemical etching of glass with aqua regia, long exposure times (6 h) of glass surfaces to concentrated hydrochloric acid [37] and nitric acid [38] were shown to effectively allow the reduction of sodium, calcium and aluminum atoms in glass by chemical etching. Our procedure, however, consisted in dipping the glass slides for under 3 min in aqua regia, which makes this an unfavorable event to take place. Nevertheless, if we consider that there was indeed a preferential chemical etching of the indentations during Au dissolution in aqua regia solution, causing them to increase both laterally and vertically, we could first correct the size of the indentations to match the one measured on the NPs to then correct the indentation depths following a similar percentage reduction. In the case of the 8 and 12 nm Au samples, this would result in subtracting approximately 10% to the NP thermal embedding values. The maximum percentage of NP thermal embedding observed in this study would then be ~77% instead of 88%, for the 12 nm Au sample annealed for 10 h 30 min.

### 3.3. Reflectivity

**Figure 4:**
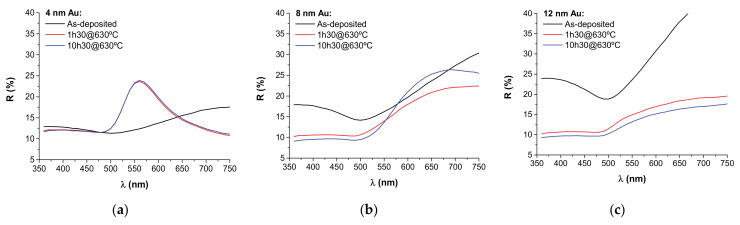
Reflectivity plots in the visible spectra of each sample before and after thermal annealing treatments at 630 °C for different times: (**a**) 4 nm Au; (**b**) 8 nm Au; (**c**) 12 nm Au.

The reflectivity spectra of the as-deposited and annealed samples are shown in Figure 4.

Concerning the as-deposited Au nanolayers, the overall reflectivity increased with the Au nanolayer thickness. For all samples the reflectivity spectra showed a stable SPR band minima centered at around 500 nm together with a reflectivity maxima at the higher wavelengths that increased in intensity with the Au nanolayer thickness. The former resonance is an intrinsic light absorption feature present in Au NPs and Au nanolayers alike (the so-called surface-enhanced bulk polaritons) [39,40]. The latter feature is indicative of vertical nanolayer growth [41].

With the annealing at 630 °C and the formation of large ellipsoidal NPs, typical LSPR scattering bands appeared in the reflectance spectra. Light absorption dominates for NP sizes below 30 nm whereas light scattering becomes important only for NP sizes above 30–40 nm, which is the case with our annealed samples. For the 4 nm Au case, a narrow LSPR scattering band appeared centered at around 558 nm, which slightly red-shifted in position with annealing time (from λ_SPR_ ≈ 557.9 nm to λ_SPR_ ≈ 559.3 nm) due to the increased refractive index surrounding the NPs (due to the slightly higher NP thermal embedding at 10 h 30 min of annealing time). This resonance is indicative of embedded ellipsoidal NPs of ~40–100 nm [25]. For the 8 nm Au case, a broad LSPR scattering band positioned at around 700 nm appeared, becoming more pronounced with the annealing time. This broad redshifted LSPR feature should be characteristic of higher aspect ratio Au islands with sizes above ~40 nm [39]. The broadening of LSPR peaks is also connected with broadened NP size distributions [3]. For this particular case (8 nm Au), this LSPR band seems to slightly blue-shift with the annealing time, which should be related with the observed morphological changes in the NPs (Figure 3b1,b2). For the 12 nm Au case this LSPR resonance is further broadened and red-shifted mostly due to the presence of larger NPs [34,39] and of broader NP size distributions [3].

### 3.4. Refractive Index Sensitivity (RIS)

**Figure 5:**
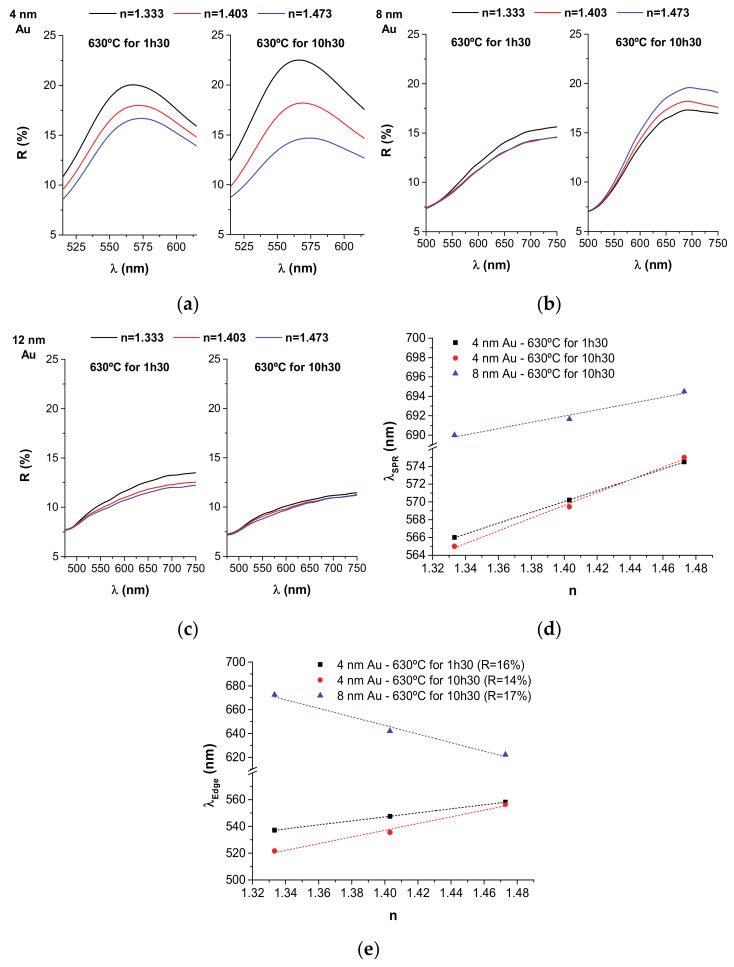
Reflectance curves of the annealed samples containing liquids with varying refractive indices: (**a**) 4 nm Au; (**b**) 8 nm Au; (**c**) 12 nm Au. The baseline of some curves in samples 8 and 12 nm Au were slightly moved up or down in intensity in order to match with the remaining curves in the lower wavelengths; (**d**) Variation of the LSPR peak position (λ_SPR_) with the surrounding refractive index (for the best samples in this study); (**e**) Variation of the LSPR band edge (λ_Edge_), for a fixed reflectance reference value, with the surrounding refractive index (for the best samples in this study). The linear trend lines are just a guide to the eye.

The reflectance curves of all samples with our test liquids of varying refractive index are shown in Figure 5a–c for samples 4 nm, 8 nm, and 12 nm Au, respectively. The evolution of the LSPR peak position with the refractive index of the surrounding medium is shown in Figure 5d for the best samples.

In the overall we have obtained good results for the samples containing sharper LSPR scattering bands, i.e., in the 4 nm Au case (Figure 5a). The RIS of the 4 nm Au samples annealed at 630 °C for 1 h 30 min and 10 h 30 min were 61 nm/RIU (or in terms of peak intensity 0.3 RU/RIU) and 72 nm/RIU (or 0.6 RU/RIU), respectively (where RIU means refractive index units and RU means reflectivity units). In the latter case, larger NPs were obtained that should be more sensitive to RI changes in the surroundings, which could compensate for the slightly higher thermal embedding. For the samples with higher Au contents, only in the case of the 8 nm Au sample annealed for 10 h 30 min (right graphic in Figure 5b) was it possible to determine the RIS, showing 32 nm/RIU (or 0.2 RU/RIU).

Our RIS results are comparable with the ones found in literature. The RIS of ion-etched sputter-deposited Au NP arrays partially embedded in quartz substrates by re-sputtering was 51–55 nm/RIU [42]. The RIS of thermally embedded Au NPs on silanized glass was 34–48 nm/RIU [23]. The RIS results of evaporated Au NPs partially embedded in glass were in the 66–153 nm/RIU range [22]. However, in this case, a lower percentage of NP thermal embedding was achieved (27%), which can partially explain the better RIS results.

The particular variations observed in the LSPR bands of our nanocomposites with the RI of the surrounding medium allows us to use another strategy for calculating the RIS, based on the spectral shift measurement [43]. In this case, we set a fixed reflectance value and then monitor the edge shift of the LSPR band (Figure 5e). By considering a fixed reflectance value of around ~14–17% (depending on the nanocomposite), we obtain RIS values of 150 nm/RIU, 251 nm/RIU and 360 nm/RIU for the 4 nm Au sample annealed for 1 h 30 min, for the 4 nm Au sample annealed for 10 h 30 min and for the 8 nm Au sample annealed for 10 h 30 min, respectively. These are quite good values for such a robust LSPR sensor using a very simple excitation/detection scheme consisting of a reflectance measurement at a fixed angle.

### 3.5. Structural Stability

The utilization of plasmonic Au NPs as LSPR optical transducers requires structural stability (namely passing the adhesive tape test or even other mechanical tests) as well as optical stability toward dipping in solvents and drying (i.e., subjecting the NPs to capillary forces that can modify or remove the NPs from the surface) [44].

All samples containing thermally embedded NPs passed successfully in the adhesive tape test. In order to further test the mechanical robustness of these samples, 1 cm viton balls were adapted into a scratch tester stylus in order to perform scratch tests using a progressive load setup up to 15 N. No plastic deformation or scratch was observed in any of the samples. This contrasted markedly with the as-deposited samples that were easily scratched. As an example, visual pictures of the scratch tested zones of the as-deposited 8 nm Au sample and of the 1 h 30 min thermally annealed 8 nm Au sample can be found in Supplementary Figure S3.

**Figure 6:**
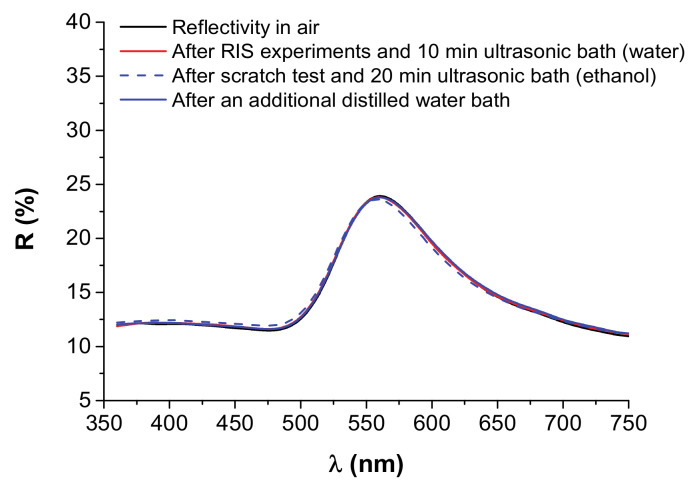
Reflectivity of the thermally annealed 4 nm Au sample (630 °C for 10 h 30 min), compared: in air; after RIS experiments using multiple RI liquids and a 10 min ultrasonic cleaning bath in distilled water; after scratch test and additional 20 min of ultrasonic cleaning bath in ethanol and drying; following an additional distilled water bath and drying procedure.

Additionally, the optical signal of the plasmonic sensors have shown excellent stability towards cyclic cleaning procedures consisting of ultrasonic cleaning bath in ethanol for 20 min and drying, which allows the robust utilization of the sensors in real world applications. An example of the optical signal before and after the RIS experiments using different RI liquids and after additional cleaning procedures can be found in Figure 6, for the 4 nm Au sample annealed for 10.5 h.

Interestingly, after ultrasonic cleaning with ethanol and drying the LSPR extinction peak is slightly blue-shifted. If an additional water bath and drying procedure is performed, the optical signal matches almost perfectly with the original signal. Therefore, this small difference in peak positioning after ethanol bath and after water bath should be related with very small amounts of H_2_O molecules that stay adsorbed at the surface of the nanocomposite, even after the drying procedure (in the case of distilled water baths), provoking a small red-shift of the LSPR extinction peak.

## 4. Conclusions

Ultra-thin Au layers, with thicknesses above the percolation limit, were sputter-deposited on glass substrates and subject to thermal annealing treatments at a temperature above the glass Tg. This process promoted dewetting, recrystallization, and thermal embedding of Au NPs of different average sizes (between 40 and 60 nm) and shapes (ellipsoids of different aspect ratios) in glass. The solid-state dewetting process of our magnetron-sputtered Au nanolayers was slower than the previously observed one in evaporated thin films, allowing for a better control over the final microstructure of the plasmonic nanomaterial. With increasing annealing time, the average NP indentation depth values increased by 14–19 nm, 38–56 nm, and 59–81 nm for the 4, 8, and 12 nm Au samples, respectively, whereas the average NP height values above the glass surface stayed approximately constant at ~12 nm. Moreover, with increasing average Au NP volume, it was observed a change in the preferential embedding orientation of the ellipsoidal NPs, from having its major axis parallel to the glass surface to having its major axis perpendicular to the glass surface. Percentages of NP thermal embedding in glass between 54% and 88% were obtained in this study, by far the largest observed in any Au–glass system.

After thermal annealing, the Au-glass samples showed strong optical scattering bands that broadened and redshifted with the Au content. Refractive index sensitivities between 150 and 360 nm/RIU (concerning LSPR band edge shift) or between 32 and 72 nm/RIU (concerning LSPR peak position shift) were obtained for the samples showing narrower LSPR extinction bands. Moreover, the thermally embedded Au NPs were highly stable, resisting both liquid immersion procedures and mechanical adhesion tests, allowing for a full-time cyclic operation of the LSPR sensor in real world applications.

## Figures and Tables

**Table 1:** Average data parameters obtained from AFM images concerning the morphologies of both Au NPs and their indentations in the glass surfaces (mean value ± standard deviation).

Sample	Au 4 nm		Au 8 nm		Au 12 nm	
Annealing Time	1 h 30	10 h 30	1 h 30	10 h 30	1 h 30	10 h 30
NP height (nm)	11.5 ± 3.4	12.6 ± 5.9	12.1 ± 16.2	11.8 ± 13.4	12.1 ± 9.8	11.6 ± 12.7
Indentation depth (nm)	13.7 ± 7.0	18.8 ± 12.2	37.5 ± 19.8	55.9 ± 29.5	58.8 ± 37.2	80.9 ± 56.4
Total NP height (nm)	25.2 ± 7.8	31.4 ± 13.5	49.6 ± 25.6	67.7 ± 32.4	70.9 ± 38.5	92.4 ± 57.8
% NP embedding	54	60	76	83	83	88
NP minor axis (nm)	38.0 ± 15.7	51.5 ± 21.4	67.6 ± 72.3	58.8 ± 66.0	36.8 ± 58.2	61.7 ± 66.2
NP Major axis (nm)	49.7 ± 28.8	70.9 ± 31.7	100.3 ± 109.4	79.7 ± 93.0	48.4 ± 83.4	75.5 ± 73.2
AR1 (top view) ^1^	1.3 ± 0.3	1.4 ± 0.3	1.5 ± 0.4	1.4 ± 0.3	1.3 ± 0.3	1.3 ± 0.2
AR2 (cross section view) ^2^	0.7 ± 0.3	0.6 ± 0.4	0.7 ± 0.9	1.2 ± 1.4	1.9 ± 3.2	1.5 ± 1.9
Indentation minor axis (nm)	30.7 ± 10.5	34.6 ± 10.5	98.8 ± 81.1	94.5 ± 58.1	96.3 ± 62.6	115.4 ± 85.9
Indentation Major axis (nm)	42.8 ± 19.2	45.5 ± 13.8	149.1 ±134.5	154.9 ± 100.3	175.9 ± 123.0	181.2 ± 141.8
AR1 (top view) ^1^	1.4 ± 0.3	1.3 ± 0.2	1.6 ± 0.4	1.6 ± 0.3	1.9 ± 1.2	1.6 ± 0.4
AR2 (cross section view) ^2^	0.8 ± 0.4	0.9 ± 0.5	0.5 ± 0.5	0.7 ± 0.6	0.7 ± 0.6	0.8 ± 0.8

^1^ AR1 is defined as the ratio between the average major and minor axis of equivalent ellipse (top-view). ^2^ AR2 is defined as the ratio between the average ellipsoidal nanoparticle total height (cross‐section) and the average minor axis (top-view) of an equivalent ellipse.

## Data Availability

The data presented in this study are available on request from the corresponding author.

## Supplementary Materials

The following are available online at https://www.mdpi.com/article/10.3390/nano11061592/s1, Figure S1: AFM images of the as-deposited nanometric thin Au layers over glass slides with varying nominal Au thicknesses, Figure S2: Distribution curves of the NP equivalent diameters for different samples, Figure S3: Color pictures of the scratch-tested 8 nm Au samples: (a) as-deposited; (b) thermally annealed at 630 °C for 1 h 30 min. The scratch tests were made using a 1 cm viton ball adapted to the scratch tester stylus, under a progressive load setup up to 15 N of maximum load. The red arrow indicates the increasing load direction.
