# Supplementary material for: Robust LSPR Sensing Using Thermally Embedded Au Nanoparticles in Glass Substrates
Source: Nanomaterials (Basel). 2021 Jun 17;11(6):1592. doi: 10.3390/nano11061592 (PMC8235226; doi:10.3390/nano11061592)
Supplement: Supplementary file 1 [file nanomaterials-11-01592-s001.zip › nanomaterials-1245882-supplementary.pdf]

nanomaterials

Supplementary Materials

Article

Robust LSPR sensing using thermally embedded Au nanoparticles in glass substrates

N.M. Figueiredo [1,*], R. Serra [1], and A. Cavaleiro [1,2]

[1] CEMMPRE - Department of Mechanical Engineering, University of Coimbra, Rua Luís Reis Santos, 3030-788 Coimbra, Portugal;

[2] IPN - LED&MAT - Instituto Pedro Nunes, Rua Pedro Nunes, 3030-199 Coimbra, Portugal.

* Correspondence: nuno.figueiredo@dem.uc.pt; Tel.: +351 239790745

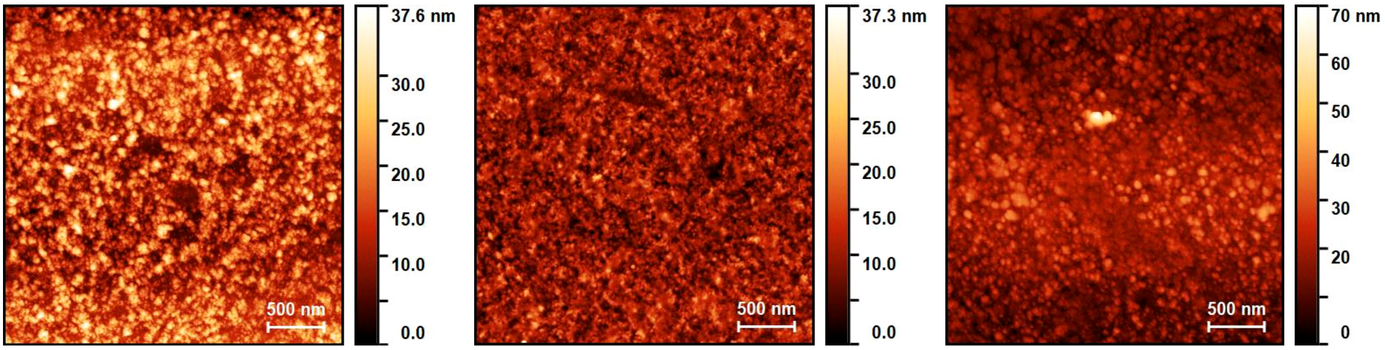

(a) (b) (c)

Figure S1. AFM images of the as-deposited nanometric thin Au layers over glass slides with varying nominal Au thicknesses: **(a)** 4 nm Au; **(b)** 8 nm Au; **(c)** 12 nm Au.

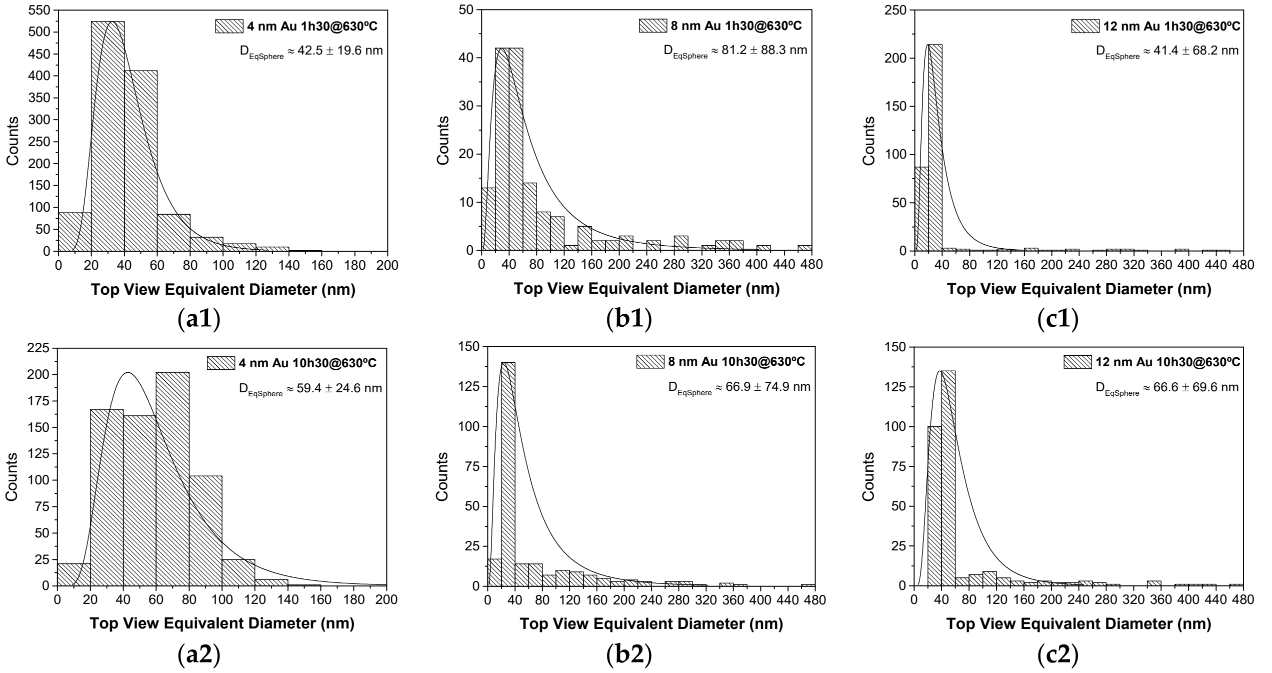

Figure S2. Distribution curves of the NP equivalent diameters for different samples: **(a1)** 4 nm Au annealed for 1h30; **(b1)** 8 nm Au annealed for 1h30; **(c1)** 12 nm Au annealed for 1h30; **(a2)** 4 nm Au annealed for 10h30; **(b2)** 8 nm Au annealed for 10h30; **(c2)** 12 nm Au annealed for 10h30.

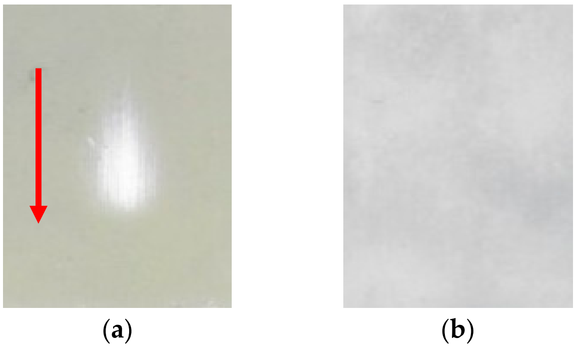

Figure S3. Color pictures of the scratch-tested 8 nm Au samples: **(a)** as-deposited; **(b)** thermally annealed at 630 °C for 1h30min. The scratch tests were made using a 1 cm viton ball adapted to the scratch tester stylus, under a progressive load setup until reaching 15 N of maximum load. The red arrow indicates the increasing load direction.
